# Epigenetics, 1-Carbon Metabolism, and Homocysteine During Dysbiosis

**DOI:** 10.3389/fphys.2020.617953

**Published:** 2021-02-23

**Authors:** Mahavir Singh, Shanna J. Hardin, Akash K. George, Wintana Eyob, Dragana Stanisic, Sathnur Pushpakumar, Suresh C. Tyagi

**Affiliations:** ^1^Department of Physiology, University of Louisville School of Medicine, Louisville, KY, United States; ^2^College of Arts and Sciences, Case Western Reserve University, Cleveland, OH, United States; ^3^Department of Dentistry, Faculty of Medical Sciences, University of Kragujevac, Kragujevac, Serbia

**Keywords:** butyrate, epigenetics, eubiosis, DNMT, MMP, REDD1

## Abstract

Although a high-fat diet (HFD) induces gut dysbiosis and cardiovascular system remodeling, the precise mechanism is unclear. We hypothesize that HFD instigates dysbiosis and cardiac muscle remodeling by inducing matrix metalloproteinases (MMPs), which leads to an increase in white adipose tissue, and treatment with lactobacillus (a ketone body donor from lactate; the substrate for the mitochondria) reverses dysbiosis-induced cardiac injury, in part, by increasing lipolysis (PGC-1α, and UCP1) and adipose tissue browning and decreasing lipogenesis. To test this hypothesis, we used wild type (WT) mice fed with HFD for 16 weeks with/without a probiotic (PB) in water. Cardiac injury was measured by CKMB activity which was found to be robust in HFD-fed mice. Interestingly, CKMB activity was normalized post PB treatment. Levels of free fatty acids (FFAs) and methylation were increased but butyrate was decreased in HFD mice, suggesting an epigenetically governed 1-carbon metabolism along with dysbiosis. Levels of PGC-1α and UCP1 were measured by Western blot analysis, and MMP activity was scored via zymography. Collagen histology was also performed. Contraction of the isolated myocytes was measured employing the ion-optic system, and functions of the heart were estimated by echocardiography. Our results suggest that mice on HFD gained weight and exhibited an increase in blood pressure. These effects were normalized by PB. Levels of fibrosis and MMP-2 activity were robust in HFD mice, and treatment with PB mitigated the fibrosis. Myocyte calcium-dependent contraction was disrupted by HFD, and treatment with PB could restore its function. We conclude that HFD induces dysbiosis, and treatment with PB creates eubiosis and browning of the adipose tissue.

## Introduction

Although metabolic syndrome is associated, in part, with a high-fat diet (HFD), the mechanistic aspect(s) of this dysregulation, and the governing of the intimate relationship or fine balance between gut eubiosis and dysbiosis, are poorly understood. In general, a balance of the gut flora (eubiosis versus dysbiosis) is normally maintained, however, during HFD consumption, this balance gets shifted to a dysbiotic state that produces harmful bacterial by-products, causing changes in the metabolism of cells lining the gastro-intestinal tract (GIT), thus affecting mitochondria and the “1-carbon metabolism (-CH3).” In brief, as previously reported by our group, during eubiosis phosphatidylcholine (PC), carnitine, trimethylamine (TMA), Hcy, methionine (Met), and dimethylglycine (DMG) are regulated by phosphatidylcholine phosphatase (PCP), along with betaine-Hcy S−methyltransferase (BHMT) in the gut (George et al., 2020). But when gut flora is perturbed (i.e., dysbiosis), the elevated levels of flavin monooxygenase (FMO) create trimethylamine N−oxide (TMAO) (which is responsible for cardiovascular stiffness) (Jiang et al., 2019), thus prompting tissue remodeling. These pathological alterations decrease the regenerative capacity of the heart (Brzezinski and Zundel, 1993). Further, host resistance to various antibiotics has been shown to be the result of inverton promoter inhibition via methylation (as induced by dysbiotic bacterial antigens), in turn, forcing the host to lose its competitive edge on harmful bacteria (Bjørndal et al., 2015; Jiang et al., 2019). Interestingly, during eubiosis, the epigenetically controlled reversible DNA methylation by DNA methyltransferase (DNMT) and phosphatidylethanolamine methyltransferase (PEMT) allows normal gene regulation activity, however, during dysbiosis the irreversible DNA methylation causes hyperhomocysteinemia (HHcy). This is responsible for cardiovascular complications, most likely as a result of the gut dysbiotic environment, leading to metabolic syndromes (Chaturvedi et al., 2014). In fact, in our opinion, the mechanism of methionine-homocysteine-betaine-cycle during eubiosis versus dysbiosis favors the epigenetically reversible and irreversible DNA methylation patterns (eubiosis versus dysbiosis) that are responsible for HHcy. The effects of a HFD dysbiotic diet on cardiac structural and functional remodeling, and its mitigation by probiotics, is central to our hypothesis in this work wherein the effects on fatty acid oxidation/lipogenesis and lipolysis might reveal the beneficial effect(s) of probiotics that could increase lipolysis and bioenergetics (Chao and Zeisel, 1990; Benakanakere et al., 2015; Bjørndal et al., 2015; Reizel et al., 2015, 2018). These above narrated metabolic changes induce shifts via modification of genes and proteins by epigenetics mechanisms, causing disruption in the epigenetic memory (imprinting and off-printing) by DNA methylation. Since global DNA methylation is used as a hallmark of epigenetic modification, interestingly, the “1-carbon metabolism” also produces homocysteine (Hcy), unequivocally (Tyagi, 1999; Ye et al., 2017). Despite all this, a balance between free fatty acids (FFAs) and the aliphatic acids, such as acetate, butyrate, and derivatives of butyrate such beta-aminoisobutyric acid (BAIBA), must be maintained. This balance is necessary during eubiosis (Roberts et al., 2014; Xie et al., 2015). Interestingly, butyrate and its derivatives decrease blood pressure and convert white adipose tissue into brown adipose tissue (Roberts et al., 2014). However, the mechanisms of HFD-induced gut dysbiosis are far from clear. Therefore, again, the hypothesis of this study is that HFD creates gut dysbiosis, increases methylation, and decreases mitochondrial bioenergetics. This causes lipogenesis. Although we previously demonstrated a decrease in matrix metalloproteinases (MMPs) by doxycycline, it is unclear whether a probiotic also mitigates MMPs activities. In this context, we opine that treatment with a probiotic mitigates hypermethylation and mitochondrial dysfunction increases browning of white adipose tissue, and improves cardiac function during intake of a high fat diet.

## Materials and Methods

### Animal Handling and Care

Male and female 8–10 weeks old wild type (WT) mice (C57BL/6J) were purchased from the Jackson Laboratory (Bar Harbor, ME, United States). The animal procedures were reviewed and subsequently approved by the Institutional Animal Care and Use Committee (IACUC) of the University of Louisville. Further, the animal care and guidelines of the National Institutes of Health (NIH, United States) were also adhered to. Experimental mice were fed with a High Fat Diet (HFD) purchased from Harlan Laboratories (Cat No. TD.88137) for 16 weeks, either with or without a probiotic (PB; *Lactobacillus rhamnosus* GG@2.5 × 10^5^ CFU) and a ketone body donor from lactate (the substrate for the mitochondria). Control mice groups were fed a standard chow diet (Table 1). All mice were allowed water *ad libitum*. Unless otherwise mentioned, three to five mice were used in each group for all experiments. Mice were treated with PB (2.5 × 10^5^ CFU), while the control mice (without PB) were given normal water. At the end of the experiment, animals were euthanized by using tribromoethanol (TBE). Mice were grouped as follows: WT; WT + HFD; WT + PB; WT + HFD + PB. The blood was collected, and the soleus and gastrocnemius muscles were isolated from each animal. The serum levels of creatine kinases (CK) activity were detected as described (Miller et al., 2000) and by using Helena Technology Kit, TX, United States.

**TABLE 1:** Composition of the high fat diet (HFD) in comparison to the standard rodent chow diet used during in the study.

**Diet Ingredients (%)**	**Standard Diet Composition (Chow)**	**High Fat Diet Composition (HFD; 42% from Fat)**
Saturated fat	4.1	61.8
Monounsaturated fat	4.6	27.3
Polyunsaturated fat	1.7	4.7
Cholesterol (g/kg)	1.7	1.5
Protein	17.2	15.2
Carbohydrate	45.2	48.5

### Blood Pressure

Blood pressure (BP) was measured in conscious mice using the non-invasive tail cuff method (CODA; Kent Scientific, Torrington, CT, United States). The animals were allowed to acclimatize to the restraining chambers on a warm platform for short periods (20–30 min) for a few days before data were recorded. Under standard conditions (room temperature, lighting, and quiet surroundings), weekly BP was recorded before and after treatment, including systolic, diastolic, and mean pressures. Complete instructions have previously been reported by our group (Pushpakumar et al., 2013; Narayanan et al., 2014; George et al., 2018).

### Antibodies

Musclin, DAAM 1, DAAM 2, and REDD 1 were purchased from Santa Cruz Biotechnology (Santa Cruz, CA, United States). DRP 1, DNMT 3B, and TFAM were purchased from Abcam (Cambridge, MA, United States), PEMT from Lifespan Biosciences, Seattle, WA, United States and the GAPDH was purchased from Millipore Sigma.

### Western Blot Analysis

Proteins from cardiac tissues were isolated using protein extraction buffer (RIPA lysis buffer, protease inhibitor cocktail and PMSF). Lysates were spun in an extraction buffer for 12 h and then centrifuged at 12,000 × *g* for 15 min. Supernatant was transferred to new tubes and protein concentrations were analyzed using the Bradford protein estimation assay. Equal amounts of total protein (50 μg) were resolved on SDS-PAGE and transferred to polyvinylidene (PVDF) membranes. The gel was transferred electrophoretically overnight onto a PVDF membrane at 4°C. The membrane was blocked with a 5% milk solution for 1 h. Primary antibodies were diluted at a concentration 1:1000 in TBST and incubated on the membrane overnight. All membranes were washed in TBST solution four times and then incubated with secondary HRP conjugated antibody solution for 2 h at room temperature. Four TBST washing steps were followed before membranes were developed using a chemiluminescent substrate in a Bio-Rad ChemiDoc (Hercules, Calif.). Band intensity was determined using densitometry analysis on Image Lab software (Bio-Rad, Hercules, CA, United States). The relative optical density of protein bands was analyzed using gel software Image Lab 3.0. The membranes were stripped and re-probed with GAPDH as a loading control.

### Beta-Hydroxybutyrate Colorimetric Assay

The beta-hydroxybutyrate colorimetric assay kit (AB83390, Cambridge, MA, United States) was used to detect beta-hydroxybutyrate abundance and the experiment was performed according to the manufacturer’s instructions. Readings were recorded at 450 nm absorbance. The colored product is proportional to the amount of beta-hydroxybutyrate abundance in the sample. We used this assay to measure the absorbance of the resulting colorimetric product using a Spectramax M2, microplate reader (Molecular Devices, San Jose, CA, United States) following 30 min of incubation in the dark.

### FFA Assay

The FFAs assay kit (MBS 779824, MyBioSource, Inc., San Diego, CA, United States) was used to detect FFAs abundance, and the experiment was performed according to the manufacturer’s instructions. Readings were recorded at 550 nm; the colored product is proportional to the amount of FFAs abundance in the sample. Absorbance of the resulting colorimetric product was measured using a Spectramax M2, microplate reader (Molecular Devices, LLC. 3860 N First Street San Jose, CA, United States).

### Global Methylation

Total genomic DNA from blood samples of the animals were isolated and analyzed for 5-methylcytidine, as described previously by our group, using the Quick-gDNA^TM^ MiniPrep kit from Zymo research (Irvine, CA, United States). After quantification, an equal amount of genomic DNA was used to estimate global levels of 5-methylcytosine using 5-mC DNA ELISA kit from Zymo research (Irvine, CA, United States) by following the manufacturer’s instructions as described earlier by our group (Narayanan et al., 2014; Veeranki et al., 2015).

### MMPs Activity

Gelatinolytic activity of MMP-2 was examined by substrate gelatin zymography. Equal amounts of proteins obtained from LV tissue homogenates were separated on a 10% sodium dodecyl sulfate-polyacrylamide gel electrophoresis (SDS-PAGE) gel containing 0.1% gelatin. The gel was washed twice with an interval of 1 h in 2.5% Triton X-100 washing buffer and then incubated in incubation buffer containing 50 mM Tris–HCl, 10 mM CaCl_2_, 1 M ZnCl_2_, and 200 mM NaCl, pH 7.5 at 37°C for 18–20 h. Then the gel was stained with Coomassie solution (0.05% Coomassie brilliant blue R-250 in 40% methanol and 10% acetic acid) and partially destained with destaining solution (20% methanol and 10% acetic acid) to visualize the clear zone of gelatin lysis against the blue background stain, indicating the presence of MMP-2. The zymography gel was imaged for the lysis zones in every lane for MMP-2 activity (Tyagi et al., 1993; George et al., 2019).

### Echocardiography

Ultrasound was performed using Vevo 2100 imaging system; cardiac and aortic data were collected as described previously (Kunkel et al., 2019). Experimental animals were placed supine on a warm platform (37°C) under isoflurane anesthesia and fixed. Using a MS550D (22–25 MHz) transducer, the thoracic cavity was imaged. Aortic arch velocity and cardiographic function were assessed in pulse wave and color Doppler modes. The transducer probe was placed on the left hemithorax of the mice in the partial left decubitus position. Two-dimensionally targeted M-mode echocardiograms were obtained from a short-axis view of the left ventricle at or just below the tip of the mitral-valve leaflet and were recorded. LV size and the thickness of LV wall were also measured. Only the M-mode ECHO with well-defined continuous interfaces of the septum and posterior wall were collected.

### Analyses of the Single Myocyte Contraction, Calcium Transit, and Cardiomyocyte Contractility Measurements

The intact hearts of experimental mice were dissected out quickly after anesthesia and perfused with freshly made perfusion buffer with Liberase TH, as described before (Tyagi, 1999; Givvimani et al., 2012). The yield was 80–85% and did not vary between the experimental groups. The isolated myocytes were used immediately for the contractility measurements, calcium transits, flow cytometry analysis, mitochondrial fractionation, and oxygen consumption rates (OCR) measurements. The systolic and diastolic functions on isolated myocytes were recorded by Ion-Optics (Boston). The decay of calcium transient was also recorded as previously described (McMillan and Hazen, 2019). A subset of cardiomyocytes were incubated with Fura-2-AM (1.0 μmol/l) for 30 min, and fluorescence measurements were recorded with a dual-excitation fluorescence photomultiplier system (IonOptix) as described before after 1 Hz field stimulation (Givvimani et al., 2012). Myocytes were field stimulated (at a frequency of 1.0 Hz, pulse duration of 4 ms and amplitude of 10 volts) using IonOptix myopacer and the contractions were recorded through SoftEdge^TM^ Acquisition Software, as described before (Givvimani et al., 2012). Randomly selected, a batch of five myocytes were recorded for the contraction parameters at a time from an unstimulated pool and a total of 20 myocyte recordings per heart were collected for further analysis.

### Histology

Cardiac tissue was preserved in plastic tissue embedding molds (Polysciences) containing tissue freezing media (Triangle Biosciences, Durham, NC, United States), were stored at −80°C, and later used for Cryosectioning. Sections of 8 μm were created using a cryocut (Leica CM 1850). Cryosections were made on superfrost plus (lysine coated) microscope slides and stored at −80°C for further immuno-histochemistry analysis.

### Statistical Analysis

All values are presented as mean ± SE from five independent experiments in all cases. Data analyses and graphical presentations were performed with the help of GraphPad InStat 3 and GraphPad Prism, version 6.07 (GraphPad Software, Inc., La Jolla, CA, United States). The experimental groups were compared by one-way analyses of variance (ANOVA) if the values were sampled from Gaussian distributions. For a set of data, if ANOVA indicated a significant difference (*p* < 0.05), Tukey-Kramer multiple comparison tests were used to compare group means. Post-test was only performed if *p* < 0.05. If the value of Tukey-Kramer ‘q’ is less than 4.046, then the *p*-value is less than 0.05 and considered statistically significant.

## Results

**FIGURE 1:**
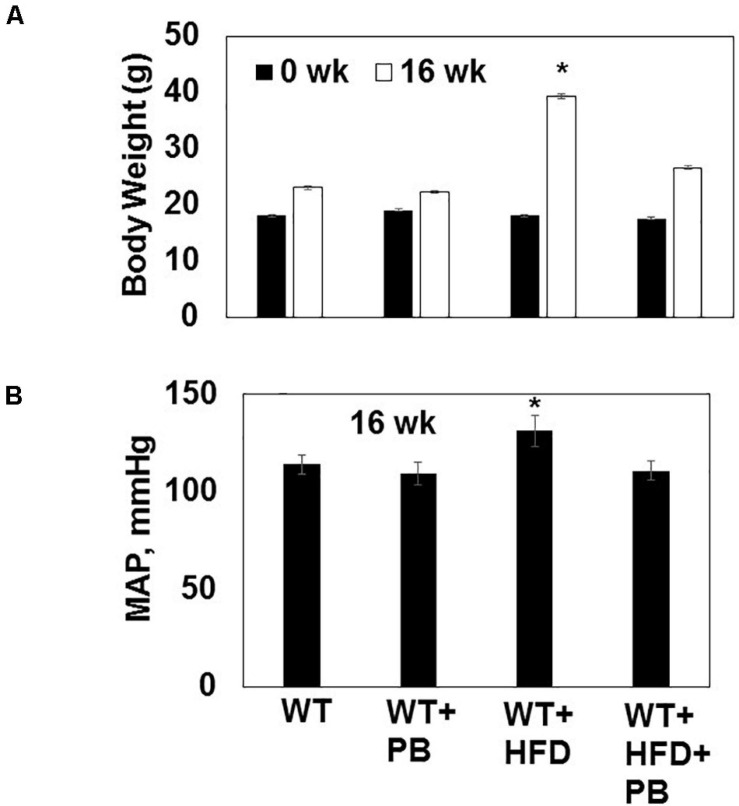
**(A)** Body weight of WT mice on a HFD with and without the probiotic treatment at the beginning (0 week) and after 16 weeks of treatment. **(B)** The mean arterial pressure (MAP, mmHg) was measured by tail-cuff and 16 weeks. *, *p* < 0.05 when compared with WT or WT + HFD + PB; *n* = 5.

**FIGURE 2:**
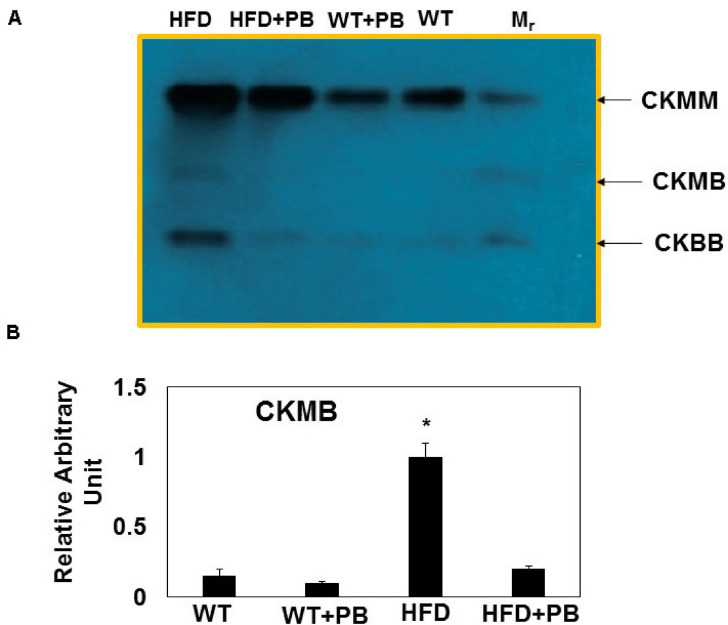
Multi-organ damage measured by estimating serum levels of: **(A)** Representative agarose gel electrophoresis of CK MM (marker of skeletal muscle injury); CK MB (marker of cardiac muscle injury) and CK BB (marker of neuronal injury); **(B)** Relative intensity of CK MB in the serum of different groups of mice. *, *p* < 0.05 when compared with WT or WT + HFD + PB; *n* = 5.

**FIGURE 3:**
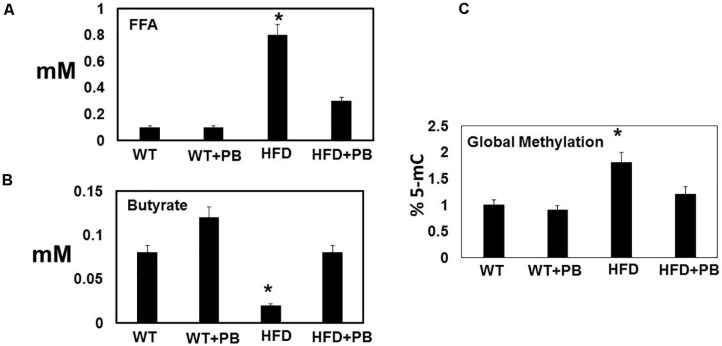
Dysbiosis induced by feeding a HFD: Serum levels of **(A)** free fatty acid (FFA); and **(B)** butyrate; and **(C)** global DNA methylation in blood borne cells. The levels were measured spectroscopically in a microplate and colorimetric methods. *, *p* < 0.05 when compared with WT or WT + HFD + PB; *n* = 5.

**FIGURE 4:**
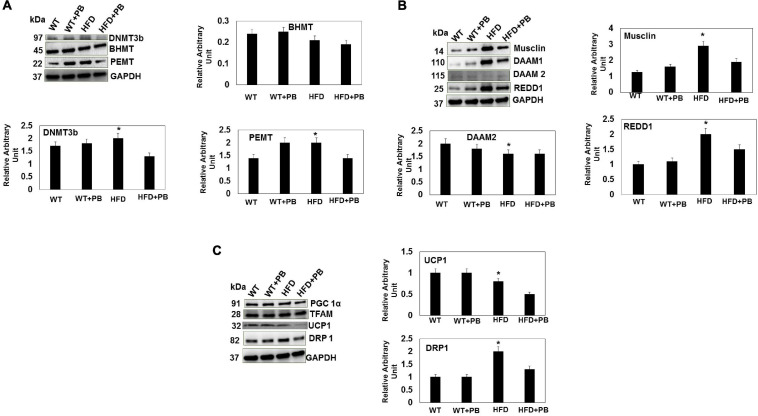
Representative Western blot analysis of DNMT, BHMT, and PEMT **(A)**; Musclin, DAAM1, DAAM2, and REDD1 **(B)**; PGC1a, TFAM, UCP1, DRP1 **(C)**, in LV tissue of WT mice on HFD with and without the treatment with PB. The bar graph represents the intensity in arbitrary unit from *n* = 5 in each group. *, *p* < 0.05 compared with WT or the WT plus HFD plus PB.

Wild type (WT) mice on HFD had gained weight significant by 16 wks. The systemic blood pressure was also increased in WT animals on a HFD diet. Interestingly, animals on a HFD plus probiotic (PB) did not gain weight and did not become hypertensive (Figure 1). Since HFD causes metabolic disorders and instigates multiple organ damage, we determined multiple organ damage by measuring creatine kinase (CK) activity specifically for the cardiac (MB), skeletal muscle (MM), and neuronal (BB) enzymes. The results suggested robust injury to cardiac and skeletal muscles. Interestingly, treatment with the PB alleviated this injury (Figure 2). To determine the levels of FFAs as a marker of dysbiosis and butyrate as a marker of eubiosis, we measured the FFAs and butyrate, respectively. The levels of FFAs were increased and butyrate was decreased in the mice on the HFD but the treatment with PB decreased the FFAs and increased the butyrate levels (Figures 3A,B). To determine the role of 1-carbon metabolism in dysbiosis, we measured total global methylation in the blood cells. The results suggested an increase in 5-methylcytidine levels in mice on a HFD and the treatment with PB mitigated the increase in 1-carbon metabolism in mice on a HFD (Figure 3C). Further, to determine the relationship between 1-carbon metabolism, methylation, and hyperhomocysteinemia (HHcy), we measured DNMT, BHMT, and PEMT. The results suggested an increase in DNMT and decrease in BHMT in HFD. The increase in PEMT was also observed. The treatment with PB decreases the levels of DNMT and PEMT, but there was no change in BHMT (Figure 4A).

To determine the role of PB in cardiac regeneration, we measured musclin, DAAM2, and REDD1. The results suggested an increase in musclin and REDD1 and a decrease in DAAM2 in the HFD group mice. Treatment with the PB normalized these levels (Figure 4B). To determine the role of mitochondrial bioenergetics, we measured the relative levels of TFAM, PGC1α, UCP1, and DRP1. Although there was no change in mitochondrial DNA and PGC1α, the UCP1 levels were decreased and the DRP1 levels were increased in mice fed with a HFD. This suggested that it was a strong indicator of change from white adipose tissue to brown adipose tissue (i.e., UCP1 levels) and fission of mitochondria (i.e., DRP1 levels) (Shimokawa et al., 2019; Mooli et al., 2020). Interestingly, the treatment with PB mitigated mitochondrial fission and promoted browning of the white adipose tissue (Figure 4C). In fact, the lactobacillus is a ketone body donor (from the lactate, the substrate for the mitochondria) that helped in a turning the white adipose tissue into the brown one.

**FIGURE 5:**
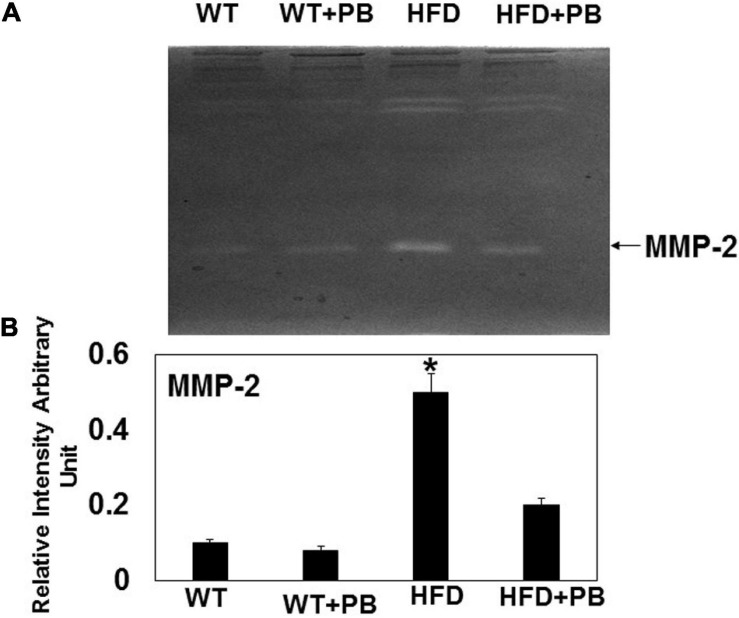
Zymography activity of MMP-2: **(A)** Representative 8%-gelatin gel zymography on cardiac tissue homogenates from WT mice on HFD with and without the treatment of probiotic. **(B)** The relative scan intensity (arbitrary unit) of MMP-2 activity. *, *p* < 0.05 when compared with WT or WT + HFD + PB; *n* = 5.

**FIGURE 6:**
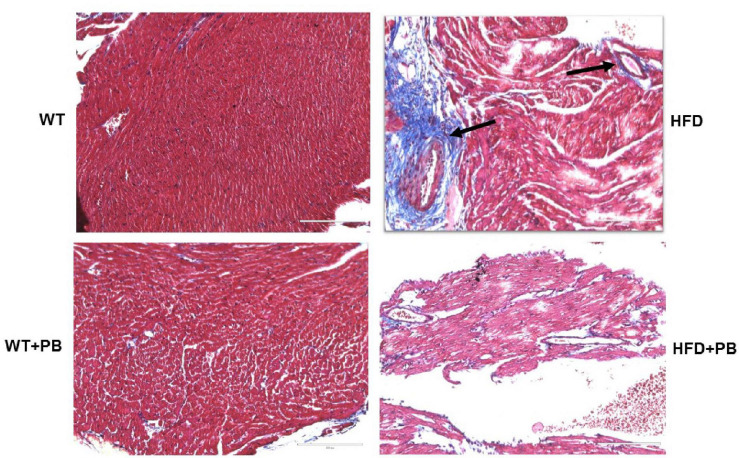
Representative Histological analysis of collagen staining by trichrome blue in the heart of WT on a HFD with and without the treatment with probiotic. Blue color represents collagen. The arrow indicates the perivascular fibrosis.

**FIGURE 7:**
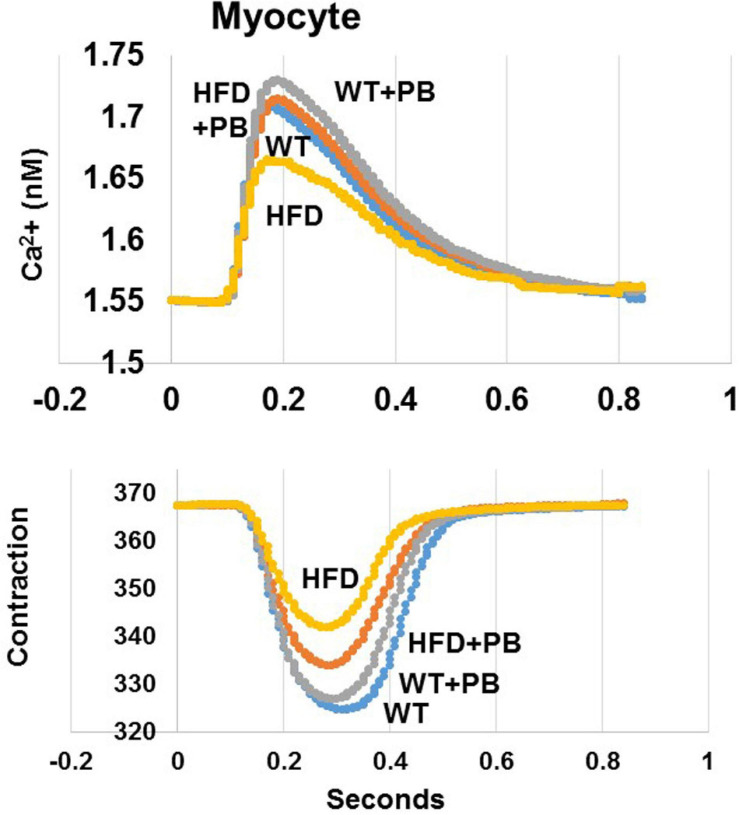
Representation of single myocyte contraction and calcium (Ca^2+^) transients as measured by Ion-Optic. Calcium transience was detected by FURA-2. The fluorescence ratio of FURA-2 and binding to calcium was recorded. Calcium ratio at cell lengthening and the duration of lengthening was measured. Myocytes were stimulated at 1 Hz. The myocyte lengthening (micron, μM) was determined as contraction in cardiomyocyte from WT mice on HFD treated with and without the probiotic, as previously reported by our group (Mishra et al., 2010).

**FIGURE 8:**
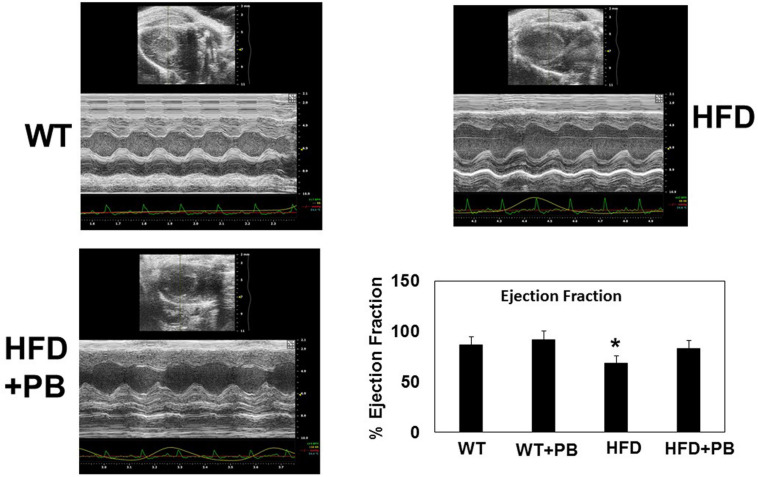
Representative scan of cardiac Echocardiography of WT mice on a HFD treated with and without probiotic. Bar graph represents the ejection fraction of the heart of mice treated with a HFD and with and without probiotic. *, *p* < 0.05 when compared with WT or WT + HFD + PB; *n* = 5.

Remodeling of an organ/tissue by its very nature implies degradation and synthesis of the extracellular matrix (ECM). In this context, we determined to measure the cardiac MMP activity. The levels of MMP-2 activity were found to be increased in the mice who were on a HFD. The treatment with PB mitigated this increase in MMP-2 activity in the HFD-fed mouse groups (Figure 5). It is important to mention that an increase in cardiac remodeling is directly associated with cardiac fibrosis. Therefore, we went for a histological evaluation of the cardiac tissue and noticed a robust increase in the perivascular as well as interstitial fibrosis in the HFD mouse groups. Interestingly, treatment with PB mitigated the fibrosis in the mice on a HFD (Figure 6). In addition to the above experiments, we studied the effects of HFD on individually isolated adult cardiomyocytes. Single myocyte contraction data revealed a dyscoordination in the calcium transient and contraction in the myocytes isolated from HFD mice; however, treatment with PB mitigated this dyscoordination in the myocytes’ calcium transient’s contraction and promoted synchronization (Figure 7 and Mishra et al., 2010). Finally, we recorded the cardiac M-mode Echo parameters. Our findings demonstrate a decrease in the ejection faction and a concomitant increase in the end-diastolic dimensions in the mice who were on a HFD. The treatment with PB was able to mitigate the decrease in ejection faction and improve the cardiac dimensions (Figure 8).

## Discussion

The results suggested an improvement in cardiac function post PB treatment during HFD consumption. The injury and remodeling were attenuated, and there were signs of regeneration in mice hearts on HFD after the treatment with PB. As previous studies have shown, an increase in the weight in mice on a HFD was associated with an increase in blood pressure (Jung et al., 2016; McMillan and Hazen, 2019). Our study revealed that PB could decrease weight and blood pressure in mice on a HFD. Although it is known that the HFD diet promotes multiple organ damage (Miller et al., 2000; Lam et al., 2012; Zhou et al., 2013), our results show that cardiac injury was robust in mice maintained on a HFD. Treatment with the PB mitigated this cardiac injury.

Our results also suggested that, although the levels of FFAs were increased, the levels of butyrate were decreased in mice eating the HFD. Others have shown that butyrate could decrease blood pressure in the hypertensive gut dysbiotic mice (Marques et al., 2017). In addition, we showed that total global 5-methylcytidine levels were increased, which were directly related with increased levels of the DNMT in mice on a HFD (Narayanan et al., 2014). At the same time, the levels of BHMT were decreased. Also, because these two experiments were performed by two different researchers, this also suggests very strong scientific rigor in our data.

Similarly, the levels of cardiac DAAM2 (factor associated with WNT-signaling) were decreased but the levels of musclin and REDD1 were increased in the HFD mouse groups. The PB could normalize the DNA damaging factor (REDD1), and thus it could improve the cardiac performance by musclin (Brzezinski and Zundel, 1993; Camp et al., 2004; Kumari et al., 2011; Mottillo et al., 2012; Hayasaka et al., 2014; Ajima et al., 2015; Subbotina et al., 2015; Obeid et al., 2016; Schwimmer et al., 2019; Zhou et al., 2019). UCP1 is associated with browning of white adipose tissue (Zhou et al., 2019). The levels of cardiac fibrosis and MMP were successfully normalized in the HFD mice treated with PB (Camp et al., 2004; Tam et al., 2014; Schwimmer et al., 2019). The discoordination in calcium transient in adult myocytes from HFD suggested the dysfunction of calcium proteins (such as SERCA and Calpain), however, treatment with the PB improved cardiac contractile function. Collectively, our results suggested that HFD induces methylation and dysbiosis, decreases butyrate, and impairs mitochondrial thermogenesis and browning of white adipose tissue, but an intervention with the PB could increase thermogenesis and the browning of the adipose tissue.

## Data Availability Statement

The raw data supporting the conclusions of this article will be made available by the authors, without undue reservation.

## Ethics Statement

The animal study was reviewed and approved by IACUC, University of Louisville School of Medicine, Louisville, KY, United States.

## Author Contributions

MS and ST conceived, designed the research, analyzed the data, edited the draft, and finalized the manuscript. SH, AG, WE, and SP performed the experiments and helped write the draft of the manuscript. All authors read and approved the final version of this manuscript.

## Conflict of Interest

The authors declare that the research was conducted in the absence of any commercial or financial relationships that could be construed as a potential conflict of interest.
